# Surveys of current status in biomedical science grant review: funding organisations' and grant reviewers' perspectives

**DOI:** 10.1186/1741-7015-8-62

**Published:** 2010-10-20

**Authors:** Sara Schroter, Trish Groves, Liselotte Højgaard

**Affiliations:** 1 BMJ Editorial, BMA House, London, UK; 2 Clinical Physiology, Nuclear Medicine & PET, Rigshospitalet, University of Copenhagen, Denmark and ESF, Strasbourg, France

## Abstract

**Background:**

The objectives of this research were (a) to describe the current status of grant review for biomedical projects and programmes from the perspectives of international funding organisations and grant reviewers, and (b) to explore funders' interest in developing uniform requirements for grant review aimed at making the processes and practices of grant review more consistent, transparent, and user friendly.

**Methods:**

A survey to a convenience sample of 57 international public and private organisations that give grants for biomedical research was conducted. Nine participating organisations then emailed a random sample of their external reviewers an invitation to participate in a second electronic survey.

**Results:**

A total of 28 of 57 (49%) organisations in 19 countries responded. Organisations reported these problems as frequent or very frequent: declined review requests (16), late reports (10), administrative burden (7), difficulty finding new reviewers (4), and reviewers not following guidelines (4). The administrative burden of the process was reported to have increased over the past 5 years. In all, 17 organisations supported the idea of uniform requirements for conducting grant review and for formatting grant proposals. A total of 258/418 (62%) reviewers responded from 22 countries. Of those, 48% (123/258) said their institutions encouraged grant review, yet only 7% (17/258) were given protected time and 74% (192/258) received no academic recognition for this. Reviewers rated these factors as extremely or very important in deciding to review proposals: 51% (131/258) desire to support external fairness, 47% (120/258) professional duty, 46% (118/258) relevance of the proposal's topic, 43% (110/258) wanting to keep up to date, 40% (104/258) desire to avoid suppression of innovation. Only 16% (42/258) reported that guidance from funders was very clear. In all, 85% (220/258) had not been trained in grant review and 64% (166/258) wanted this.

**Conclusions:**

Funders reported a growing workload of biomedical proposals that is getting harder to peer review. Just under half of grant reviewers take part for the good of science and professional development, but many report lack of academic and practical support and clear guidance. Around two-thirds of funders supported the development of uniform requirements for the format and peer review of proposals to help ease the current situation.

## Background

Peer review for submissions to scientific journals has developed over more than 300 years, and there is now a considerable body of evidence on its methods, outcomes, effectiveness, best practice, problems, and ethics [1]. Peer review of applications for research grants has a sparser evidence base and fewer examples of good practice for funders to draw on when developing their systems. This lack of evidence is worrying, because several major funders of biomedical research have reported recently that they are becoming overburdened by workloads and by the complexity and slowness of grant review processes [2,3].

In contrast, editorial peer review at journals has a much wider evidence base and is subject to continuous review and debate. Strengths, weaknesses, ways to reduce bias, and different models in journal peer review have been studied extensively, yet many questions remain unanswered. Although there is evidence on how to conduct peer review fairly and efficiently, there is only limited evidence that journal peer review improves the quality of published biomedical science according to a Cochrane review that systematically reviewed 28 of 61 retrieved studies [4]. International congresses have been held regularly since 1989 to present and share the latest evidence and to develop further research questions, so that biomedical journal editors can try to improve their practices [5]. Additionally, several international organisations gather, further develop, and promote evidence to guide editors; particularly the International Committee of Medical Journal Editors (ICMJE) whose Uniform Requirements for Manuscripts Submitted to Biomedical Journals guidelines ensure the consistency and transparency of manuscripts and guide practice, processes and policies at more than 800 journals [6]. There is, however, no equivalent resource for preparing grants and conducting grant review.

There has been one Cochrane systematic review about grant review practices, but it found evidence only on biases and other process weaknesses and included only 10 studies [7]. The authors concluded 'We were unable to find comparative studies assessing the actual effect of peer review procedures on the quality of the funded research. There is little empirical evidence on the effects of grant giving peer review. Experimental studies assessing the effects of grant giving peer review on importance, relevance, usefulness, soundness of methods, soundness of ethics, completeness and accuracy of funded research are urgently needed. Practices aimed to control and evaluate the potentially negative effects of peer review should be implemented meanwhile' [7]. Until the necessary studies of grant review are done, might funders develop and use a set of uniform requirements, at least partly informed by the evidence on journal peer review?

Ernest Starling was an eminent physiologist who when asked by the British Medical Research Council in the 1920s about how to best distribute funding answered 'get the best of men, give them the equipment you can afford, and leave them alone'. Peer review is the main 'equipment' used by research councils and other funders. To explore the current status of grant review, the eponymous 'Starling Group' of funders, policy makers, researchers, and editors, who first met in Frankfurt to discuss the European Medical Research Councils' strategy for medical research in Europe [3], initiated two surveys.

The first Starling Group study is an international survey to describe the current status of peer review among biomedical funding organisations and the problems they face when evaluating proposals for biomedical project and programme grants. The second is a survey to determine the workload of external grant reviewers, the level of institutional support for this activity, reviewers' motivations and perceived barriers to taking on grant review, and their views on possible solutions. We report on both surveys here.

## Methods

### Survey of biomedical funding organisations

#### Sample

We took a convenience sample of biomedical research funding organisations across Europe and also approached key national funders from North America, Australia, and New Zealand to broaden the survey's reach and relevance; members of the Starling Group suggested international public and private grant giving organisations they thought should be included. We also sought the advice of the European Foundation Centre for suggestions for inclusions of private foundations in Europe. The final list of 57 funding organisations included both small and large international funding organisations. The purpose was to include a range of different funders from different countries to illustrate some of the current problems they face rather than to create a representative sample and draw inferences beyond the sample.

#### Procedures

A draft questionnaire was developed based on discussions with several funding organisations about current practice and common problems with peer review. The questionnaire was then refined by members of the Starling Group and revised before field testing. The questionnaire asked participants to respond to all questions in relation to grants that provide support for biomedical studies addressing a single research question or research theme (project and programme grants) not infrastructure grants or fellowships. We sent an invitation and link to the electronic survey on SurveyMonkey (online survey software; http://www.SurveyMonkey.com) to a named contact person at the funding organisation explaining the purpose of the research and requesting their help. Non-responders were sent an email reminder 2 weeks and 6 weeks after the original mailing.

### Survey of external grant reviewers

#### Sample

Funding organisations that took part in our first survey were invited to survey a random sample of approximately 50 of their reviewers. Nine organisations agreed to take part and emailed invitations to our survey to a small sample of their external reviewers.

#### Procedures

A draft questionnaire was developed based on known problems with grant peer review and previous research with journal peer reviewers [3]. The draft questionnaire was then finalised by members of the Starling Group. Reviewers were sent an email from their funder informing them of the survey with a link to the questionnaire on SurveyMonkey. Reviewers were informed that the survey was being conducted for research purposes by a team interested in improving grant review processes and that their responses would be confidential. All reviewers were emailed a reminder by their funding organisation to complete the survey approximately 2 weeks after the initial mailing.

## Results

### Survey of biomedical funding organisations

#### Sample characteristics

We received a response from 29 (52%) of the 56 funders. Table 1 is a list of participating organisations. Organisations were based in 19 countries, were funded by various sources, and varied in size and the amount of grant money administered. Six large organisations took part. These receive over 1000 proposals a year, and were: the US National Institutes of Health, the Canadian Institutes of Health Research, The Wellcome Trust, the National Health And Medical Research Council (of Australia), the Deutsche Forschungsgemeinschaft (DFG), and the Medical Research Council, UK. Of the 29 responding organisations, 2 do not use external reviewers for project applications but use committees instead (The Swedish Research Council and The Lundbeck Foundation) and for this reason, they were excluded from the analysis of specific questions regarding external reviewers.

**Table 1:** List of participating funding organisations

Organisation	Country	Funding basis
National Institutes of Health* (NIH)	USA	Government

Canadian Institutes of Health Research*	Canada	Government and small amount of support from donations; additional funds through partnerships with private and public sector agencies

Vetenskapsradet-Medicine (Swedish Research Council)	Sweden	Government

Fonds voor Wetenschappelijk Onderzoek (FWO)	Belgium	Private foundation and research council

The Health Foundation	UK	Independent foundation

Health Research Board of Ireland	Ireland	Government

The Netherlands Organisation of Health Research and Development (ZonMw), also on behalf of the Netherlands Organisation of Scientific Research (NWO)	The Netherlands	Government

Swiss National Science Foundation	Switzerland	Private foundation

NETSCC, part of National Institute for Health Research (NIHR), in England	UK	Government

Estonian Science Foundation	Estonia	Government

The Research Council of Norway	Norway	Government (all the ministries)

Robert Bosch Stiftung	Germany	Private foundation

Foundation for Polish Science	Poland	Private foundation

Fondation Fournier Majoie pour l'Innovation	Belgium	Private foundation

National Institute for Health Research Central Commissioning Facility, which runs four grant funding schemes on behalf of NIHR: Research for Patient Benefit, Invention for Innovation and Research for Innovation, Speculation and Creativity (all project grants) and Programme Grants for Applied Research	UK	Government

The Wellcome Trust*	UK	Charity

National Health And Medical Research Council (of Australia)*	Australia	Government

Fondazione Cariplo Milano	Italy	Private foundation

Telethon Foundation Italy	Italy	Charity

Juvenile Diabetes Research Foundation	USA	Charity

Health Research Council of New Zealand	New Zealand	Government

Medical Research Council, UK*	UK	Government

Deutsche Forschungsgemeinschaft* (DFG)	Germany	Government and private foundation

Hungarian Scientific Research Fund (OTKA)	Hungary	Government

Academy of Sciences of the Czech Republic. Grant Agency of the Academy of Sciences of the Czech Republic	Czech Republic	Government

Lundbeck Foundation, Copenhagen	Denmark	Charity, private foundation, commercial foundation

Fondation Mérieux	France	Private foundation

Fonds National de la Recherche Scientifique	Belgium	Private foundation

The Danish Medical Research Council	Denmark	Government

*Indicates large organisations (receiving over 1000 proposals a year).

#### Peer review process

A total of 15/29 of the organisations reject 10% or less of research project grant applications based on internal review only. However, 13/29 of the organisations accept 30% or less of project grant applications following external review. A total of 17/27 use an electronic tracking system to contact external reviewers and manage their reviews but few (6/29) use an electronic system for grant applicants to track their proposals during each stage of the decision-making process.

#### Selection of external reviewers

Only 13/27 organisations limit the number of annual requests to external reviewers in order to reduce individual burden and the potential for bias in the system. However, 18/27 organisations invite more than 3 external reviewers on average to review a single application. In all, 16/27 use programme managers, 15/27 boards/panels/committees, 6/27 board chairpersons, and 10/27 grant applicants to suggest external reviewers.

#### External reviewer guidelines and forms

The majority (22/27) provide guidelines for external reviewers on what applications should be judged on, 22/27 provide review forms or templates for external reviewers to submit their reviews, and 21/27 a scoring/ranking system to rate specific aspects of proposals.

#### Transparency of review process

Only 2/27 organisations hide the grant applicants' names from the external reviewers whereas 21/27 hide external reviewers' names from applicants. Only 4/27 hide the names of all reviewers on the funding board/panel from applicants. However, 5/27 organisations commented that they make a list of reviewers' names available (for example, on their website) at the end of the funding round.

During the review process, 7/27 organisations allow grant applicants to see the full external reviewers' reports, 3/27 the funding board/panels' comments/reports, and 5/27 the scores assigned to their application. However, after the decision has been made on their application, these figures rose to 16/27, 18/27, and 13/27, respectively. A total of 22/27 organisations routinely ask their reviewers to declare their conflicts of interest for each proposal reviewed.

#### Frequency of problems with specific aspects of peer review

Table 2 shows the frequency organisations reported they experience problems with specific of aspects of peer review. Problems reported as very frequent or frequent by at least a quarter of the organisations included reviewers declining to review (16/29) and receiving late reports (10/29). None of the organisations reported very frequent or frequent problems with having an inadequate number of reviewers' reports available at time of assessment, reviewers not declaring their conflicts of interest, or reviewers breaking confidentiality.

**Table 2:** Frequency organisations experience problems with specific aspects of peer review and perceived change over time

	Frequency of problem, n (%)					Perceived change, n (%)			
	
	Never	Occasionally	Frequently	Very frequently	Not applicable	Better situation now than 5 years ago	No change	Worse situation now than 5 years ago	Not applicable
Reviewers declining to review	0 (0)	9 (31)	14 (48)	2 (7)	1 (3)	2 (7)	9 (31)	13 (45)	3 (10)
Difficulty finding new reviewers for your database system	3 (10)	15 (52)	4 (14)	0 (0)	4 (14)	4 (14)	10 (35)	5 (17)	8 (28)
Difficulty retaining good reviewers	4 (14)	17 (59)	1 (3)	0 (0)	3 (10)	3 (10)	14 (48)	4 (14)	5 (17)
Having an inadequate number of reviewers' reports available at time of assessment	2 (7)	23 (79)	0 (0)	0 (0)	1 (3)	4 (14)	15 (52)	3 (10)	5 (17)
Receiving poor quality reviews	1 (3)	22 (76)	3 (10)	0 (0)	0 (0)	5 (17)	19 (66)	0 (0)	3 (10)
Receiving late reviewers' reports	0 (0)	16 (55)	10 (35)	0 (0)	0 (0)	4 (14)	19 (66)	2 (7)	3 (10)
Reviewers not following guidelines appropriately	2 (7)	20 (69)	4 (14)	0 (0)	0 (0)	5 (17)	20 (69)	0 (0)	3 (10)
Reviewers not declaring their conflicts of interest	9 (31)	16 (55)	0 (0)	0 (0)	1 (3)	7 (24)	16 (55)	0 (0)	3 (10)
Reviewers breaking confidentiality	9 (31)	12 (41)	0 (0)	0 (0)	5 (17)	1 (3)	16 (55)	0 (0)	8 (28)
Applicants questioning the conflicts of interest of reviewers	11 (38)	13 (45)	1 (3)	0 (0)	1 (3)	2 (7)	18 (62)	1 (3)	5 (17)
Applicants questioning the funder's choice of reviewers	4 (14)	18 (62)	1 (3)	1 (3)	2 (7)	2 (7)	20 (69)	0 (0)	5 (17)
Applicants recommending inappropriate reviewers	3 (10)	11 (38)	4 (14)	0 (0)	7 (24)	18 (62)	0 (0)	0 (0)	9 (31)
Difficulty in recruiting younger reviewers	9 (31)	8 (28)	2 (7)	1 (3)	6 (21)	6 (21)	11 (38)	2 (7)	6 (21)
Difficulty in recruiting female reviewers	7 (24)	9 (31)	2 (7)	1 (3)	7 (24)	5 (17)	12 (41)	1 (3)	6 (21)
Too many grant applications in the system	6 (21)	12 (41)	4 (14)	1 (3)	3 (10)	2 (7)	9 (31)	12 (41)	5 (17)
Administrative burden of dealing with peer review process	2 (7)	15 (52)	5 (17)	2 (7)	2 (7)	1 (3)	13 (45)	11 (38)	3 (10)

#### Perceived change in specific aspects of peer review over the last 5 years

Over half the sample perceived no change over the last 5 years in the following problems: having an inadequate number of reviewers' reports available at time of assessment (15/29), receiving poor quality reviews (19/29), receiving late reviews (19/29), reviewers not following guidelines appropriately (20/29), reviewers not declaring their conflicts of interest (16/29), reviewers breaking confidentiality (16/29), applicants questioning the conflicts of interest of reviewers (18/29), and applicants questioning the funder's choice of reviewers (20/29) (Table 2).

Many organisations reported the situation was worse now than 5 years ago for reviewers declining to review (13/29), too many applications in the system (12/29), and the administrative burden of process (11/29). Funders reported a better situation now than 5 years ago for applicants recommending inappropriate reviewers (18/29) reviewers declaring their conflicts of interest.

#### Most important challenges facing funding organisations

The self-reported most important challenges faced by organisations included finding available and suitable reviewers, problems with review quality and time taken to complete reviews, and problems with administration and transparency.

#### Reviewer incentives: feedback, acknowledgement, and rewards

In all, 14/27 organisations routinely give external peer reviewers the funding board/panel's decision on the proposal(s) they reviewed, but only 3/27 the details of their discussions and decisions. Only about a quarter (7/27) give feedback to reviewers on the usefulness of their reviews.

Some organisations reward or acknowledge their reviewers by naming them on their website (7/27), telling their institutions that they are reviewers for their organisation (4/27), giving feedback on the quality of their reviews (5/27), and by paying them (10/27). Other ways of thanking them included: naming them in the annual report, annual letters of thanks, informing those that give the most useful reports that they are the best, honorariums, giving formal confirmation if requested, invitations to the grant delivery ceremony or inauguration dinners, emails of gratitude and feedback on reviews if requested, allowing reviewers to submit grant applications outside of call deadlines, reviewers' award programs, and dinner invitations.

#### Development of standards on grant review

We proposed that a set of standards, such as the ICMJE guidelines [4] for the preparation and formatting of manuscripts submitted for publication by biomedical journals, drawn up for grant review might help enable researchers, funders, and peer reviewers to practice grant review more efficiently and effectively. We found considerable support for the idea of the development of a set of standards for the peer review process at grant giving organisations: 17/29 would support this idea, none would not, and 11/29 were unsure. There was also good support for the development of a set of standards for the grant application process: 17/29 would support it, 2/29 would not, and 9/29 were unsure.

### Survey of external grant reviewers

#### Sample characteristics

In all, 9 funding organisations took part and we received an overall response of 258/418 (62%) (Table 3). Two-thirds of respondents were male, and the majority (62%) was aged 41 to 60 years. In all, 77% described themselves as researchers, 31% practicing clinicians, and 17% laboratory scientists. Respondents were working in 22 countries and ranged in their experience of reviewing: 24% had been a grant reviewer for biomedical science proposals for ≤3 years and 47% for at least 10 years. Many respondents were reviewers for several funding organisation; 44% (114/258) had reviewed for 3 or more organisations in the past 12 months.

**Table 3:** Participating funding organisations and the response rates

Organisation	Country	Number of surveys sent out*	Number of surveys completed	Response rate (%)
Fonds voor Wetenschappelijk Onderzoek (FWO)	Belgium	41	19	46
Deutsche Forschungsgemeinschaft (DFG)	Germany	49	32	65
National Health And Medical Research Council (of Australia)	Australia	50	42	84
Hungarian Scientific Research Fund (OTKA)	Hungary	50	36	72
NIHR Coordinating Centre for Health Technology Assessment (NCCHTA)	UK	50	22	44
Estonian Science Foundation	Estonia	50	38	76
Telethon Italy	Italy	49	24	49
The Research Council of Norway	Norway	31	19	61
National Institute for Health Research Central Commissioning Facility	UK	48	26	54
Total		418	258	62

*After correcting for email delivery failures.

#### Time to review

Reviewers were asked to indicate approximately how long it takes them to review a single biomedical science grant proposal (including time reading proposal, making notes, writing comments, completing assessment sheets, attending committees and panels). Only 15% (39/258) said that the whole process took them less than or equal to 3 hours; 30% (77/258) spend on average between 4 and 6 hours, 9% (22/258) spend 7 to 9 hours, 17% (44/258) spend 10 to 24 hours and 10% (27/258) spend more than 24 hour.

#### Institutional support

A total of 48% (123/258) said their institution or managers encouraged them to take part in biomedical science grant review, yet only 14% (37/258) said their institution or managers knew how much time they spent reviewing and 31% (79/258) knew which funding organisations they reviewed for. A total of 32% (82/258) were expected to review grants in their own time (that is, out of office hours) and only 7% (17/258) were given protected time to conduct grant review. In all, 28% (73/258) said they always conduct biomedical science grant review in their own time, 44% (113/258) often do, 19% (49/258) occasionally and only 1% (2/258) never do.

#### Academic recognition

A total of 74% (192/258) do not receive any academic recognition for conducting grant review. Comments from the 43 who said they did included the fact that it contributes to promotional review, is recognised within the 'indicators of esteem' element of the UK's Research Assessment Exercise, is one of the metrics for assessing research portfolios in clinical departmental review, is a recognised research service when applying for grants and fellowships, and prestige.

#### Importance of specific factors in decision to do grant review

Reviewers rated the following as extremely or very important in their decision to review: 51% (131/258) to help external fairness in decision taking by review committees, 47% (120/258) sense of professional duty, 46% (118/258) relevance of the topic, 43% (110/258) wanting to keep up to date on research advances, 40% (104/258) to help ensure innovation is not suppressed (Table 4). In all, 30% (77/258) said the most important motivating factor was a sense of professional duty, 14% (35/258) the opportunity to learn something new, 14% (35/258) to keep up to date on research advances, and 12% (31/258) wanting to help external fairness in decision taking.

**Table 4:** Importance of specific factors in reviewers' decisions to conduct review

	Not at all important, n (%)	Slightly important, n (%)	Important, n (%)	Very important, n (%)	Extremely important, n (%)	Most important factor, n (%)
Opportunity to learn something new	5 (2)	46 (18)	98 (38)	70 (27)	24 (9)	35 (14)
Wanting to keep up to date on research advances in specific areas	7 (3)	39 (15)	88 (34)	85 (33)	25 (10)	35 (14)
Relevance of the topic to your own work or interests	5 (2)	35 (14)	85 (33)	91 (35)	27 (11)	25 (10)
Wanting to enhance your CV and career prospects	85 (33)	98 (38)	37 (14)	20 (8)	3 (1)	6 (2)
Reputation of the funding organisation	25 (10)	65 (25)	104 (43)	41 (16)	7 (3)	2 (1)
Wanting to get known as a reviewer	94 (36)	65 (25)	60 (23)	16 (6)	4 (2)	4 (2)
A sense of professional duty	9 (4)	19 (7)	93 (36)	82 (32)	38 (15)	77 (30)
Wanting to help pay back the efforts of others	18 (7)	49 (19)	92 (36)	67 (26)	16 (6)	10 (4)
Wanting to help external fairness in decision taking by grant review committees	8 (3)	22 (9)	82 (32)	88 (34)	43 (17)	31 (12)
Wanting to help ensure innovation is not suppressed	9 (4)	40 (16)	87 (34)	70 (27)	34 (13)	7 (3)
Wanting to keep your reviewing skills up to date	49 (19)	62 (24)	77 (30)	41 (16)	11 (4)	1 (1)

Percentages do not sum to 100% due to rounding and/or missing data.

#### Training and guidance in grant review

Only 9% (22/258) had received some formal training in how to conduct biomedical science grant review and 64% (166/258) said they would be interested in receiving training if funding organisations provided it (free of charge). Whilst 63% (162/258) reported that instructions and guidance for external reviewers provided by biomedical science funding organisations are quite clear and that they usually know what they are expect to do as a reviewer, only 16% (42/258) said that these were very clear and they always know what they are expected to do. Only 9% (23/258) reported that the clarity of instructions and guidance varied by organisation.

#### Barriers to reviewing

At least 25% of the reviewers reported the following factors often or always acted as barriers to undertaking grant review: conflicts with other workload (47%, 121/258), having to review too many journal articles (36%, 93/258), reviewing taking too much time (33%, 85/258), insufficient knowledge on the focus of the application (31%, 80/258), tight deadlines for completing the review (28%, 71/258), and having to review too many grant proposals for funding organisations (25%, 64/258) (Table 5).

**Table 5:** Frequency of specific factors cited by reviewers as barriers to undertaking grant review

	Never, n (%)	Sometimes, n (%)	Often, n (%)	Always, n (%)	Not applicable, n (%)
Insufficient interest in the focus of the application	32 (12)	152 (59)	39 (15)	8 (3)	7 (3)
Insufficient knowledge on the focus of the application	14 (5)	136 (53)	50 (19)	30 (12)	9 (4)
Having to review too many grants for funding organisations	46 (18)	114 (44)	54 (21)	10 (4)	12 (5)
Having to review too many journal articles	38 (15)	99 (38)	74 (29)	19 (7)	8 (3)
Long grant applications	67 (26)	104 (40)	47 (18)	10 (4)	9 (4)
Poor quality of the grant applications	67 (26)	107 (42)	38 (15)	12 (5)	11 (4)
Reviewing taking too much time	35 (14)	111 (43)	68 (26)	17 (7)	5 (2)
Inadequate guidance on the requirements for review	116 (45)	98 (38)	15 (6)	2 (1)	7 (3)
Believing that there is a more appropriate reviewer for the application	32 (12)	162 (63)	33 (13)	6 (2)	5 (2)
Tight deadlines for completing the review	36 (14)	125 (48)	59 (23)	12 (5)	4 (2)
Conflicts with other workload	25 (10)	85 (33)	81 (31)	40 (16)	6 (2)
Lack of formal recognition of reviewer contributions	134 (52)	56 (22)	25 (10)	12 (5)	6 (2)
Not being paid for reviewing	141 (55)	52 (20)	19 (7)	13 (5)	9 (4)
Not being paid enough for reviewing	160 (62)	28 (11)	16 (6)	7 (3)	24 (9)
Having conflicting interests with the applicants	68 (26)	126 (49)	17 (7)	20 (8)	7 (3)
Your own success rate with the funder	163 (63)	50 (19)	7 (3)	1 (1)	14 (5)
Lack of fluency in the language in which the proposal is written	172 (67)	35 (14)	3 (1)	4 (2)	23 (9)

Percentages do not sum to 100% due to rounding and/or missing data.

#### Suggestions on how funding organisations might better identify and retain grant reviewers

We solicited suggestions on how the problem of some funding organisations finding it difficult to both identify new and retain current grant reviewers might be improved. We received numerous constructive suggestions and grouped these by broad themes. Recurring themes included the need for: public acknowledgement, some kind of reward or reimbursement, institutions to recognise the importance of reviewing as an activity, tips for making reviewing easier, more internal reviewing/screening of applications, improved guidance and training, improved feedback and communication, improved administration, greater transparency, funding of more applications, an expanded reviewer pool, tips to actively solicit reviewers, and some suggestions for alternative review systems.

## Discussion

Our survey of funding organisations showed that funders are challenged by an increasing number of applications and frequent difficulty in finding willing and available reviewers. Some organisations emphasised the need for streamlining and more efficient administrative systems and we found support for the idea of uniform requirements for proposals. The survey of external reviewers showed that reviewers are motivated by a sense of professional duty and fairness despite not receiving academic recognition and frequently having to undertake this time-consuming work in their own time. Reviewers also indicated that they are often sent requests for reviews outside of their expertise suggesting that funding organisations are having difficulty targeting the appropriate reviewers. We also identified a need for improved guidance and training on how to review. These findings are similar to the problems journals experience with peer review [1].

This international survey included a diverse range of funding organisations and identified common problems. We are unaware of any other studies attempting to collect data from funding organisations with such breadth and geographical reach, although individual organisations, particularly the US National Institutes of Health (NIH) [8], have conducted their own internal reviews. We acknowledge that 29 organisations is still a small number of grant giving organisations, that we used convenience sampling which could have introduced bias, and that the response rate to this survey was low (52%). Similarly, our reviewers' survey is the first large survey of external grant reviewers from a range of funding organisations. We asked reviewers about their reviewing practices in general rather than isolated experiences with specific funders, to make the results more generalisable to the wider population of reviewers and funders. We achieved a good response rate (62%) from the reviewers similar to the response rate we have achieved with surveys of journal peer reviewers [9].

The ICMJE set an editorial precedent for the development of uniform requirements. A similar set of guidelines on grant review might enable researchers, funders, and peer reviewers to practise grant review more consistently and, we hope, more efficiently and effectively. Most of the funding organisations in the survey were receptive to the idea of such standards for grant review and reviewers indicated problems with heterogeneous requirements from funders. Further exploration of the feasibility and acceptability of uniform requirements for grant review is required.

With a bursting system reliant on good will, is it time for all funding organisations to more formally recognise contributions from reviewers through, for example, public acknowledgement, certificates, or rewards? Funding organisations should help reviewers to do their job effectively by providing clear guidance and training as well as improved feedback and communication. We are not the first to advocate the need for nurturing reviewers [10], but most have focused on journal peer review and grant review seems relatively neglected.

## Conclusions

The workload of biomedical research funders is growing. Our surveys suggest that few funders have used their experiences of deteriorating efficiency or the (albeit limited) evidence base on editorial peer review to assess and improve their processes. We suggest that funders provide clearer guidance to reviewers, draw on evidence from both editorial and grant review to maximise the efficiency and fairness of their work, and come together to consider the development of a set of uniform requirements for submitting and peer reviewing biomedical grant proposals.

## Competing interests

SS has no competing interests. TG is a member of Council of the Committee on Publication Ethics, which produces guidance on ethics aspects of peer review and directly advises editors on handling difficult submissions. LH is Chair of EMRC, the European Medical Research Councils, ESF, Strasbourg, EMRC is the membership organisation of the public funders in medical research in Europe. LH is a frequent peer reviewer of grant proposals.

## Authors' contributions

SS helped develop the content of the questionnaires, conducted the survey, analysed the results, and wrote the first draft of the manuscript. TG and LH initiated the research, helped develop the content of the questionnaires, and helped write the manuscript. All authors read and approved the final manuscript.

## Pre-publication history

The pre-publication history for this paper can be accessed here:

http://www.biomedcentral.com/1741-7015/8/62/prepub
